# High-frequency oscillatory ventilation use in patients with H1N1: a single-centre review

**DOI:** 10.1186/cc13480

**Published:** 2014-03-17

**Authors:** N Smith, N Pathmanthan, V Martinson, J Glazebrook, R Rao, L Sleight, A Gratix

**Affiliations:** 1 Hull and East Yorkshire NHS Trust, Hull, UK

## Introduction

Limited evidence exists as to the value of high-frequency oscillatory ventilation (HFOV) for patients with H1N1 [1][2]. We describe a subgroup of patients, from a single UK centre, who received HFOV and had H1N1-positive virology.

## Methods

Local permissions for research were obtained. Patients with confirmed H1N1 who underwent HFOV between 2008 and 2012 were included. Data collected retrospectively included demographics, diagnosis, illness severity and outcome measures.

## Results

We identified 10 patients (Table 1) who received both HFOV and had confirmed H1N1. Two patients were transferred to a tertiary centre to receive ECMO and both critical care and 6-month mortality were 40%.

**Table 1:** Patient characteristics

Male%	60
Age (years)	39.4 ± 9.75
Mean APACHE II	19.5 ± 4.88
Pre-HFOV neuromuscular blockade infusion (%)	40
Pre-HFOV vasoactive drug infusion (%)	50
Mean pre-HFOV VT exp (ml/kg)	8.54 ± 2.68
Median P/F ratio (mmHg)	
Pre HFOV	67.5
0 hours	94.35
4 hours	110.4

## Conclusion

This study adds to the literature on the use of HFOV in H1N1 patients. We managed to replicate some of the existing evidence in respect to the population age [1][2] and the incidence of mortality. Whilst P/F ratios improved on initiation of HFOV, these patients subsequently had long critical care and hospital stays. There is uncertainty regarding the use of HFOV in ARDS, but it may be a valuable treatment for H1N1 patients. The ventilation strategies employed and the subsequent consequences for H1N1 patients require further evaluation.
